# Improvements in the learnability of smartphone haptic interfaces for visually impaired users

**DOI:** 10.1371/journal.pone.0225053

**Published:** 2019-11-11

**Authors:** F. J. González-Cañete, J. L. López Rodríguez, P. M. Galdón, A. Díaz-Estrella

**Affiliations:** Department of Electronic Technology, University of Malaga, Campus de Teatinos, Malaga, Spain; Università degli Studi di Perugia, ITALY

## Abstract

We report the results of a study on the learnability of haptic icons used as alerts or notifications in smartphones. The aim was to explore the feasibility of using haptic icons to create assistive technologies for people with visual impairments. We compared the performance and satisfaction of users with different visual capacities (visually impaired vs. sighted) and using different learning processes (with or without a reinforcement learning stage). The reinforcement learning stage improves the recognition rate in both types of users, although the improvement obtained by the visually impaired users is even better as their recognition rates become very similar to those obtained by the sighted users. Finally, it was observed that the better recognized haptic icons are those assigned to the most employed applications by the user.

## Introduction

Research on haptics for smartphones has demonstrated that the use of vibrotactile stimuli is an excellent way to transmit complex information [1]. Recently, high-resolution haptic feedback systems for mobile phones have become prevalent, allowing the transmission of rich information through the haptic channel. For example, the TouchSense technology created by Immersion has been recently deployed in LG smartphones [2]. Similarly, Apple devices such as the iPhone 6 s and newer iPhone models as well as the Apple Watch and MacBooks from 2015 implement the Taptic Engine as part of their Force Touch technology [3].

Initially, most vibrotactile stimuli used in typical mobile phones transmitted very simple information, such as alerts. However, modern operating systems such as iOS allow the assignment of vibration sequences (predefined or user defined) to contacts in the agenda or to native services. A similar functionality can be achieved in Android-based smartphones using applications such as Good Vibrations [4]. This application allows the configuring of vibration patterns not only for different applications but also for specific contacts.

Adding complex vibrotactile stimuli to smartphones is a key advantage for users with some type of sensory deficiency, such as users that are deaf or visually impaired (VI). This technology allows improved communication using the sense of touch to compensate or even substitute for deficiencies in other senses [5][6]. In addition, the vibrotactile stimuli also increase privacy, as the vibrations are only perceived by the user. These stimuli also improve the user’s experience as they employ an additional channel for communication apart from the standard ones (audio and visual channels), and in some cases, such as in loud environments (e.g., a concert), they could substitute for the audio channel [7].

To communicate complex information, the vibrotactile stimuli need to be associated with specific meanings. These meaningful tactile stimuli are called tactons (tactile icons) or haptic icons. Tactons are defined as structured abstract messages that can be used to communicate messages non-visually [8]. Haptic icons use a metaphorical approach to establish an association between the vibrations and the concept [9].

In a previous study [10], an application (ConTactos) was presented that associated the haptic icons and the vibration pattern using a metaphor. For instance, the vibration associated with the contact called ‘partner’ was a heartbeat, and the vibration of the contact called ‘neighbours’ was a knocking door. This kind of metaphor is not used here because the current work allows associating the haptic icons with the alerts freely and not in a predetermined way.

Vibration signals are mainly employed as a way to transmit alert information as they have the capability to attract the user’s attention even in loud environments. Consequently, they are ideal for notifications such as for incoming calls or meetings. These notifications are usually composed of a simple vibration to attract the user’s attention but without any other type of related information.

The human cognitive system imposes potential limitations for the use of haptic icons [11,12], and hence, their design should allow the information-processing limitations to be clearly perceived and their meaning easily learned [13][14][15].

### Design of haptic icons for mobile phones

Currently, some tools and development kits allow for the easy development of haptic icons. Android Haptics [16] is a section of the Material Design Guidelines, Components and Tools that support the best practices of user interface design for the Android operating system. Texas Instruments has developed the TI Haptics technology [17]. It is composed of a set of hardware actuators and drivers that are controlled and configured by the Haptic Control Console software.

### Learnability of haptic icons

Learnability is one of the key aspects when designing and implementing haptic icons. Consequently, understanding the process of learning the meaning of a set of haptic icons has been an important issue in haptic icon research [18]. From the beginning of the use of computer graphical interfaces, there has been a set of designed icons that are now easily recognized and identified by users. However, icons are not inherently meaningful, and users must learn the link between the stimulus and its meaning. For instance, the save icon is usually represented with a floppy disk, although young people have probably never used one. However, they learn that this icon is employed to save even though there is not a clear correspondence for them about the meaning of the image. The link between the stimulus and its meaning can range from representational to abstract. Some icons are purely representational, linking objects and their meanings. For instance, a tweet sound could indicate the reception of a message from the Twitter application. Other icons are abstract without intrinsic meanings but are associated with arbitrary relationships that have to be learned, such as Windows error sounds [19].

To learn the association between a haptic icon and its meaning, in the work of Enriquez et al [20], a three-stage approximation was proposed. The first stage is a self-guided learning process in which users learn the association between the haptic stimuli and its meaning. In this stage, the haptic stimuli are presented along with a representation of their meaning. This representation can be a textual, audio, and/or a graphical description. The second stage is based on reinforcement; in other words, the user must recognize the associated meaning of a haptic stimulus and receive feedback on whether the answer is correct. The last stage is the same as the second stage but without feedback.

In the study of Adam Swerdfeger [21], a unimodal learning process was employed in which a textual description was displayed on the screen next to a button. This button triggers the associated haptic stimulus when pressed. However, multimodal learning processes are advantageous, as stated in several learning theories such as the dual coding theory [22] or the multimedia learning theory [23].

In recent years, game dynamics have been employed to obtain better results in the learning process [24]. This technique is named gamification and can also be applied to help visually impaired people learn to use a smartphone. The usage of games to improve learning capacity is a line of research currently in development [25]. In the work of [26], a gamified mobile application, oriented toward elderly or mildly visually impaired people, was implemented. In [27], a videogame named Audio Haptic Maze was designed, implemented and evaluated. It was oriented toward blind people and used sounds as well as haptic interfaces to guide the user to the exit of a maze. An application that assists visually impaired children in learning to type words was presented in [28], while the work of [29] developed an application for reinforcing the binary calculation skills of engineering students.

### Haptic abilities of visually impaired people

For non-visually impaired people, haptic icons can support (or substitute if desired) the visual sensation; meanwhile, for visually impaired people, haptic icons can provide them with an alternative form of sensory information. Indeed, as the number of VI people who use mobile technologies increases [30] several applications that use haptic icons to communicate information have been developed, including an assistive technology that uses the haptic channel to give feedback to the user being guided [31].

It has been noticed that visually impaired people outperform sighted people in terms of recognition when using the haptic information due to cognitive-processing differences [32]. Some authors have provided empirical evidence, from a neurophysiological perspective, stating that people without the ability to see can compensate for this by enhancing their other senses, that is, sensory compensation [33]. For instance, significant sensory differences have been identified between visually impaired and sighted people in terms of their tactile sensory threshold [34]. In addition, some studies [12] [35] [36] have demonstrated that visually impaired people have better memorization skills and show better recognition performance at haptic tasks than sighted people.

### Objectives of the study

In this study, a mobile application has been designed to assist users in learning the association between a set of vibrotactile stimuli and the alarms and notifications of a set of commonly used mobile applications. This application uses gamification and a reinforcement stage to improve the learning process.

Based on the literature reviewed, our hypotheses were as follows:

- A reinforcement stage could improve the recognition rate as well as the subjective perception and distinction of the haptic icons.
- The use of haptic icons can benefit visually impaired users.
- The use of haptic icons associated with smartphone application alerts improves the user experience.
- Both improvements also apply to visually impaired users.

## Materials and methods

### Participants

Forty-six participants of ages between eighteen and sixty performed the experiments. Eighteen of the participants were visually impaired so that they could not use graphical user interfaces, and twenty-eight of them were fully sighted non-visually impaired users. All of them were smartphone users, those with vision problems used applications such as the Android Accessibility Suite as a way to interact with their smartphones. This suite is usually preinstalled in Android-based smartphones.

The data obtained were analysed anonymously and the study was reviewed and approved by the Experimentation Ethic Committee of the University of Málaga. They are all available from S1 File.

### Experimental design

This study followed a 2x2 design, taking the user’s visual capacity (VI vs. non-VI) and the learning process (with and without reinforcement) as independent variables. The dependent variables were the time taken to answer the test, the proportion of recognized haptic icons and the results obtained by a usability questionnaire.

In the reinforcement learning scenario, some participants performed a previous learning stage in which they had to pair haptic icons with the application alerts. They received feedback informing them if their answer was right or wrong. This stage was named learning reinforcement.

Half of the VI subjects performed the reinforcement stage as well as half of the non-VI subjects. Consequently, nine VI and fourteen non-VI subjects executed the reinforcement stage and nine VI and fourteen non-VI subjects did not use reinforcement.

### Stimuli and devices

An Android application named HAPP (Haptic APPlication) has been developed to perform this study. Its source code can be accessed online (https://github.com/Equinoxe-fgc/HAPP). HAPP implements a set of sixteen haptic icons each of which is associated with an application alert for the smartphone. The haptic icons have been designed using the Haptic Studio tool [37], and they follow rhythmic patterns with a short duration (2–4 seconds) to easily memorize and distinguish them. Hence, they do not follow any predefined meaning or relationship with the application they represent.

The main parameters to take into consideration for the design of haptic icons are the frequency, amplitude, waveform and duration of the vibrotactile stimulus [38] [39]. However, haptic icons must be designed to be easily recognized, and hence, a more complex combination of those parameters can be employed. Musical techniques are useful for obtaining sets of identifiable haptic icons that are distinguishable from one another. For instance, rhythm, defined as a group of pulses of different durations and amplitude, have been proven to be easily remembered [40]. In addition to rhythm, other musical techniques have been applied to create sets of haptic icons, such as the use of crescendo, defined as a steady increase in force or intensity [41] and melody, defined as a variation in rhythm, frequency and amplitude [21].

**Fig 1:**
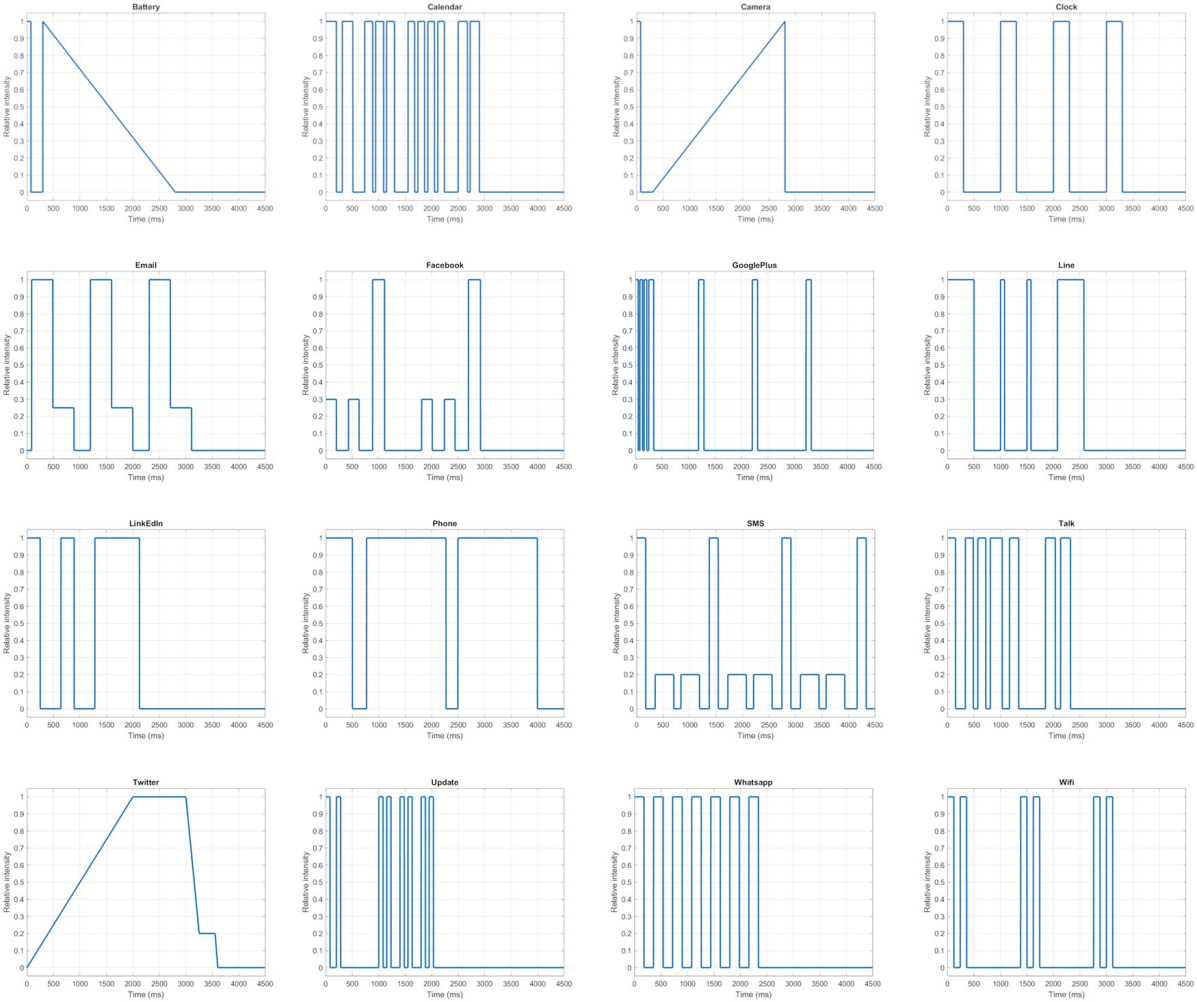
Vibrating patterns associated with the haptic icons used by the HAPP application. X-axis represents the time in milliseconds and the Y-axis represents the relative intensity of the vibration. A zero value denotes no vibration meanwhile a one value is the maximum vibration intensity.

Fig 1 shows the vibration pattern for the haptic icons used by the HAPP application.

The Y axis represents the intensity of the signal from 0 to 1, with a value of 0 representing the absence of a vibration and a value of 1 representing the maximum vibration intensity. The X axis represents the time, in milliseconds, that the vibration lasts. Most of the patterns use combinations of full vibration intensity and no vibration. However, the E-Mail, Facebook and SMS applications also employ lower vibration values (between 0,2 and 0,3). The Battery and Camera applications use sforzandos, defined as a high intensity vibration followed by a silence and a crescendo or decrescendo in the intensity of the vibration. The use of sforzandos in the design of haptic icons was studied in [41] where it was concluded that they should not be used because they caused confusion and were not clearly differentiated. Nevertheless, this work did not consider the effect of training, and it is proposed as future work. Finally, Twitter uses the ASDR (Attack-Sustain-Decay-Release) technique, where the vibration magnitude increases from 0 to the highest level (Attack), and it is maintained for a certain amount of time (Sustain). Afterwards, the vibration intensity decreases (Decay) and is maintained again to finally decrease to 0 (Release).

To evaluate the distinctiveness of the designed haptic icons, we performed a multidimensional scaling study. The selected vibration attributes are as follows:

- Number of pulses: defined as the number of vibrations between the absences of vibration.
- Number of repetitions: The number of times a pattern is repeated.
- Duration: The time that a vibration lasts in seconds.
- Softness: This parameter indicates whether the vibration has any crescendo or decrescendo (value 3) or not (value 1).
- Sharpness: Similar to softness, this parameter indicates whether the vibration has any abrupt change from no vibration to high vibration (value 3) or not (value 1).
- Crescendo: The Battery and Camera have the same vibration characteristics except for the use of a crescendo or decrescendo for the intensity of the vibration. Hence, this parameter indicates whether the vibration has a crescendo (value of 3) or not (value of 1). In addition, the Twitter haptic icon has both a crescendo and decrescendo, although the crescendo time is longer than the decrescendo time.
- Two-level intensity: A value of 3 indicates that the vibration has two different levels of intensity; otherwise, the intensity is the highest.
- Frequency dispersion: This parameter defines the variation in the frequency of the vibration. It is calculated as the quotient between the maximum and minimum periods of the vibration.

**Fig 2:**
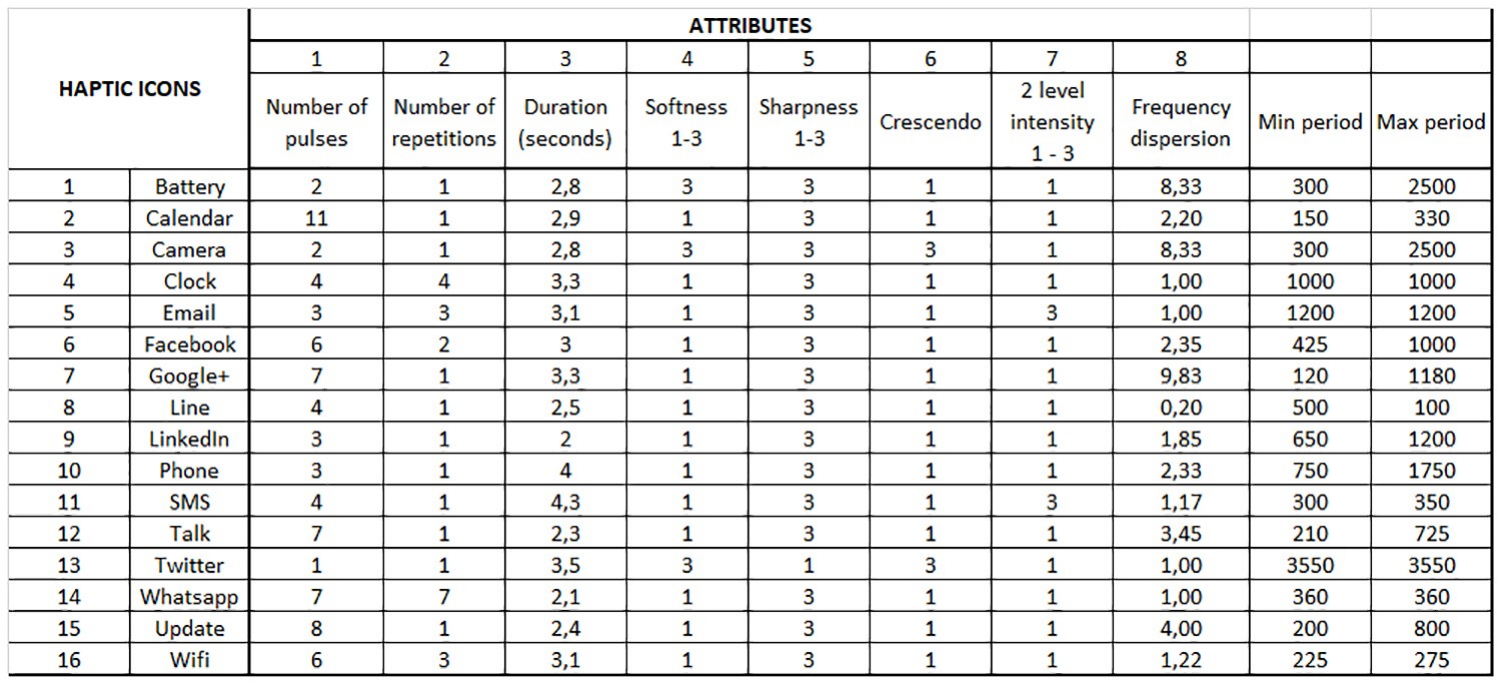
Multidimensional scaling attributes. The figure shows the selected values of the eight attributes used to perform the multidimensional scaling study of the vibration patterns.

We consider the selected parameters to be sufficient to characterize the difference between the vibration patterns. The values obtained to develop the study are shown in Fig 2.

**Fig 3:**
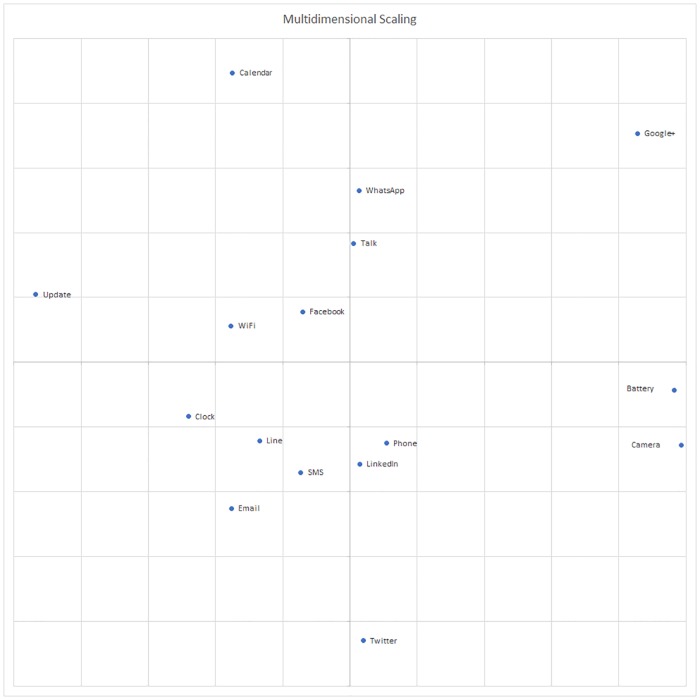
Multidimensional scaling representation of the vibration characteristics of the designed haptic icons. Short distances between icons denotes similarity; on the contrary long distances between icons represent dissimilarity.

We used the Euclidean distance to perform a multidimensional scaling study and obtained a stress value of 0,01, which indicates a good fit as it is very close to zero. The representation of the multidimensional scaling is shown in Fig 3.

It can be observed that the distance between the haptic icon vibrations is enough to be differentiated. The Update, Calendar, Google+ and Twitter haptic icons as well as those for the pair composed of Battery and Camera are very distinguishable as they are very far from each other. On the one hand, the Update, Calendar and Google+ haptic icons employ a rhythmic pattern that is not used in the rest of the haptic icons. On the other hand, the Battery haptic icon employs a decrescendo, the Camera haptic icon employs a crescendo and the Twitter haptic icon employs both a crescendo and decrescendo.

Apart from the mathematical study of vibration distinguishability, we have also performed a perceptual similarity map study with five subjects to confirm the distinguishability assumption. Each subject had to use the haptic icons in pairs and fill out a perceptual table in which each position represents the distinguishability of a pair of icons. The selected values range from 1 to 5. A value of 1 indicates that the two vibration patterns are almost undistinguishable. In contrast, a value of 5 indicates that the vibration patterns compared are clearly distinguishable.

**Fig 4:**
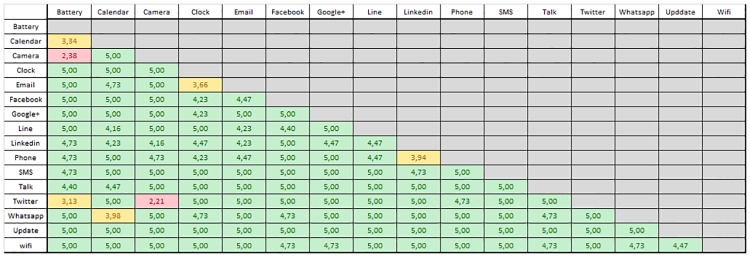
Perceptual similarity map of the haptic icon vibration patterns. Values greater than 4 represent low similarity; values ranging from 3 to 4 denote medium similarity and values lower than 3 represent high similarity.

Fig 4 depicts the obtained perceptual similarity map where each value is the mean of the values selected by the five users. All the obtained perceptual similarity maps are available in S1 File.

Values greater than 4 are shown with a green background, indicating that the vibration pair is easily distinguishable. Values ranging from 3 to 4 are depicted using a yellow background. Values lower than 3 are shown in red, indicating that they could be difficult to distinguish.

The pairs Battery-Camera and Camera-Twitter obtained the lowest values; these are the haptic icons that use a crescendo and/or decrescendo. The Battery haptic icon uses a short vibration pulse and a decrescendo, while the Camera haptic icon uses a short vibration and a crescendo both with the same duration. It seems that people have difficulty in differentiating these vibrations, as was studied in [41], although the multidimensional scaling representation showed that the vibrations are distinguishable. Nevertheless, these icons will not be discarded as we are going to study the influence of the reinforcement learning process as suggested by [42]. Surprisingly, the Camera-Twitter haptic icon pair is shown to be very far apart in the multidimensional scaling representation, and apart from the Twitter haptic icon, it is the only one that has an ADSR vibration pattern; these icons present the lowest perception value. Despite the apparent recognition problems obtained, we decided to maintain the initial haptic icon design as the mathematical distinguishability test obtained very different results.

The HAPP application implements visual icons, haptic icons, and audio described help to be usable by visually impaired people. The usage of screen read tools such as TalkBack was rejected as they interfere with the application usability. Hence, an interface that emulates the help provided by TalkBack has been developed for visually impaired people.

The application also implements an event log to register the time of all the touches performed by the user while the application is in use. The event log records the instant when an event is triggered (touch an icon) as well as the icon touched. This allows for the deduction of the usage speed and the success ratio when the icons are paired.

### Experimental procedure

Prior to the sessions, the participants were instructed to memorize the association between a set of sixteen vibrotactile stimuli and their associated application. The experiment should determine if the usage of complex vibrations obtains a good success ratio relative to the capacity to learn the association between the haptic icons and the application alerts and if the usage of a reinforcement stage improves this success ratio. In addition, the experiment should also determine whether both improvements apply to visually impaired people, and if so, whether they could be employed to improve the smartphone accessibility for people with visual disabilities.

Following the learning process proposed by [20], the application usage was divided into three stages: two learning stages and one stage where the subjects filled in a form designed to determine how easily they remembered the associations between the haptic icons and the application alerts.

**Fig 5:**
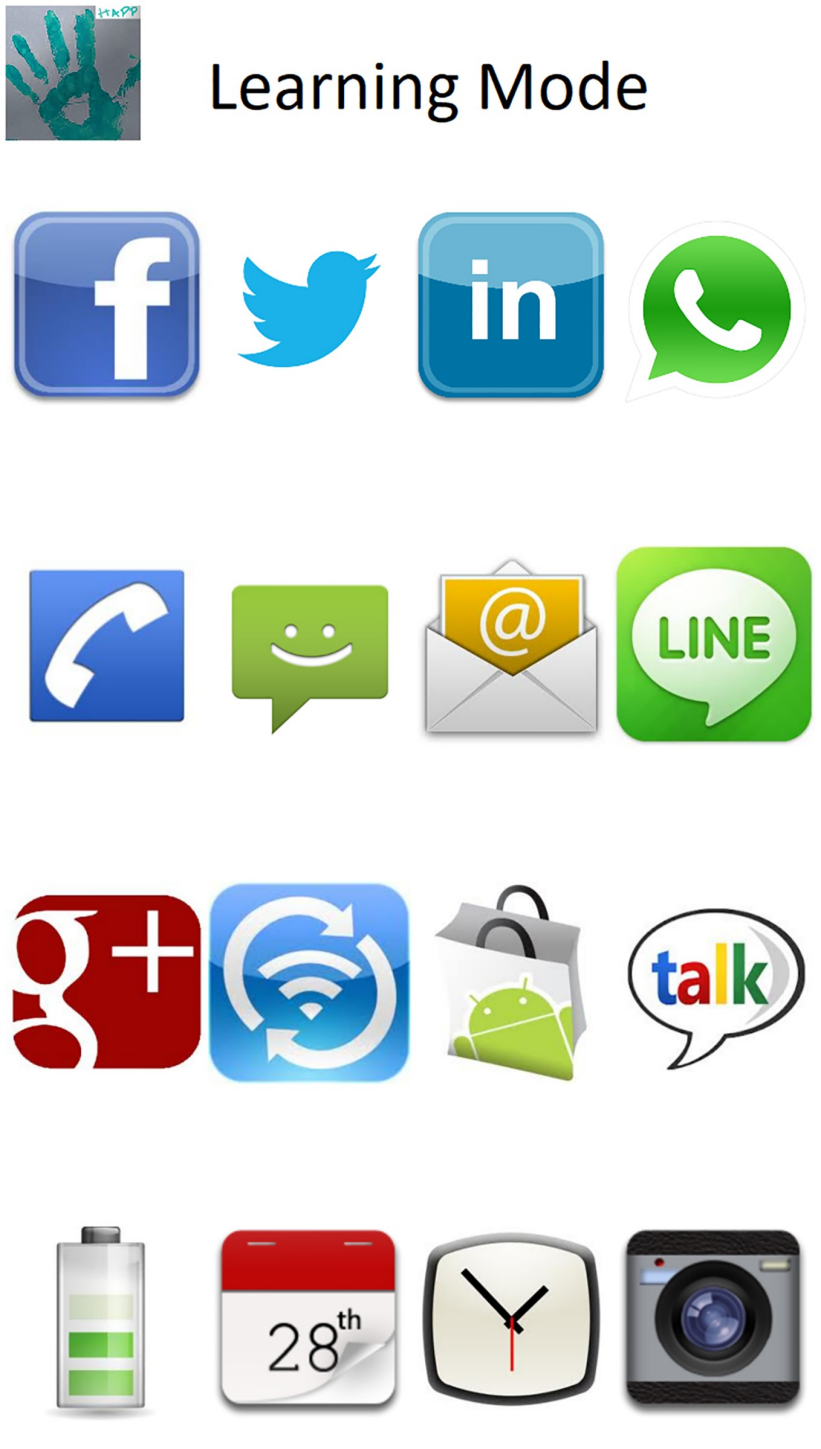
Screen capture of the learning stage of the HAPP application. In the learning mode users feel the vibration of each selected haptic icon and listen to the icon description.

**Fig 6:**
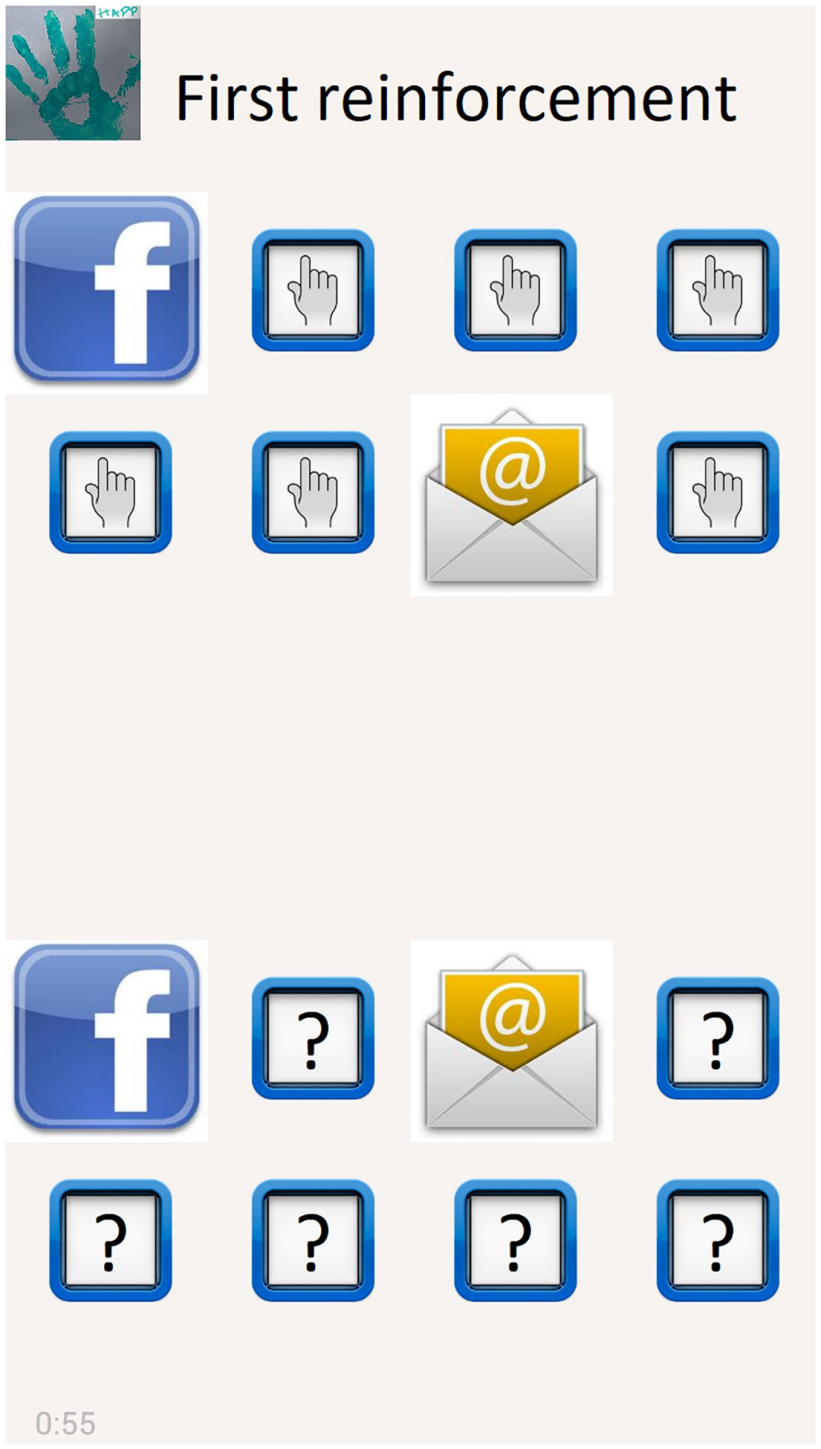
Screen capture of the reinforcement learning stage of the HAPP application. Users must pair the applications with their corresponding haptic icons. Feedback is given to inform whether the pair is correct.

**Fig 7:**
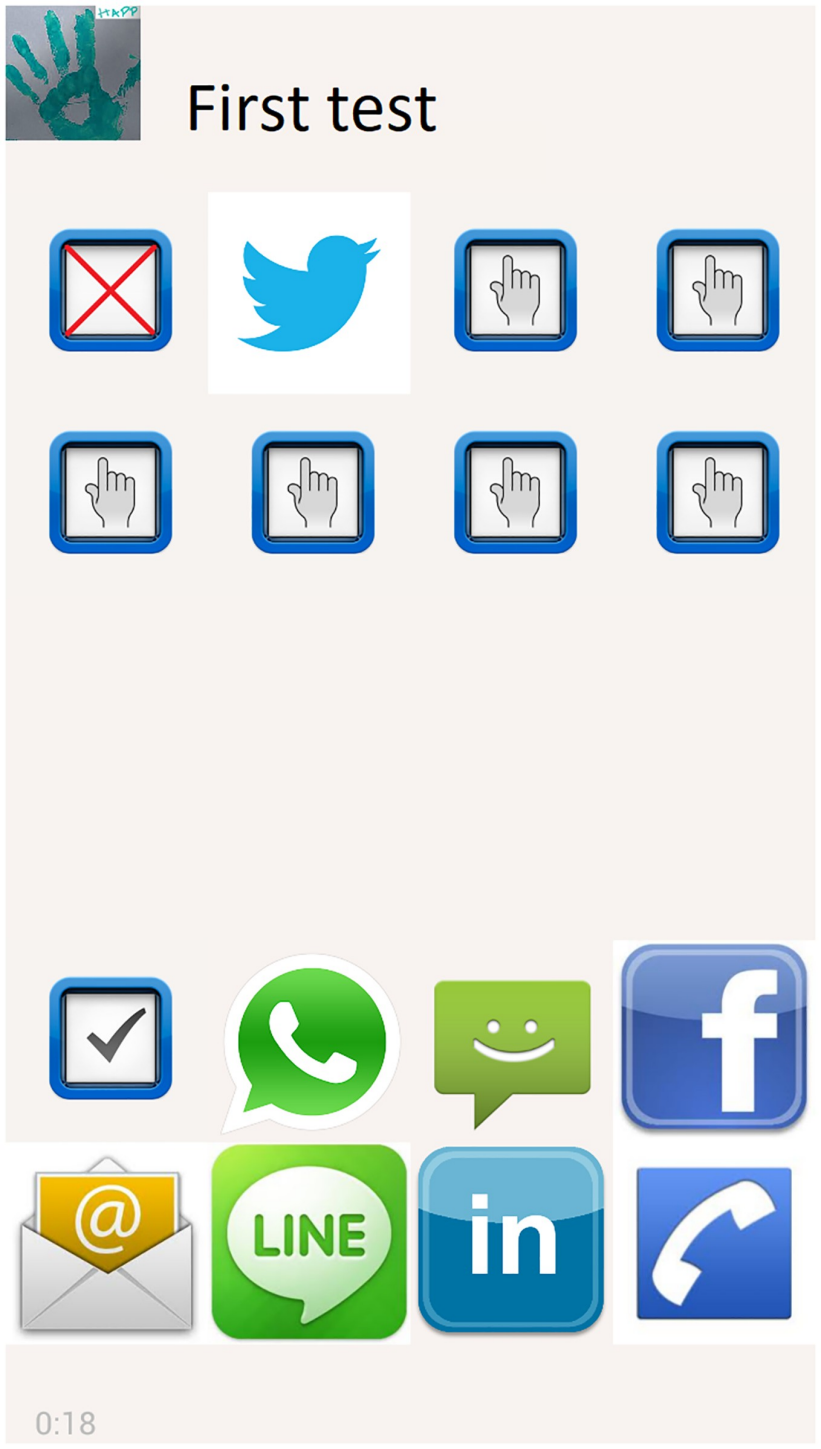
Screen capture of the test stage of the HAPP application. This stage tests if users are able to remember the pairs of vibrations and applications.

The usage stages are as follows:

- In the first learning stage, sixteen haptic icons are shown to the subject for their memorization. In this stage, the subject explores the smartphone screen, and a recorded description associated with each application is played when the subject touches the haptic icons. When a haptic icon is pressed, the smartphone plays the vibration associated with the haptic icon of this application. Fig 5 shows the screen for this learning stage of the application.
- In the reinforcement learning stage, the subject plays a game with the aim of improving the recognition of the haptic icons. The objective of the game is to pair the applications with their corresponding haptic icon as in the pair card game. In this stage, two screens with a grid of the sixteen icons were shown to the user. The eight icons located at the top of the screen vibrate when they are pressed according to their related haptic icon. The eight icons located at the bottom of the screen are the application icons, and they are initially hidden by a question mark (?) symbol. When a pair of icons are pressed (one of the top group and one of the bottom group), a voice says the name of the application and whether the pairing is correct. If the pairing is correct, the application icon will be shown face up as in the card game, and a voice saying the name of the application will be played when it is pressed again. If the pairing is wrong, the icons are hidden again. To be usable by people with a visual disability, different feedback sounds (beeps) are played depending on the zone (top or bottom zone), where the screen is pressed (Fig 6).
- The test stage has the objective of checking whether the subjects are able to remember the association between the haptic icons and the applications. This stage shows two screens with a grid of sixteen icons, and the subject has to pair the haptic icons with their applications as fast as possible. As in the reinforcement stage, the screen is divided into two sections. The section located at the top of the screen contains eight haptic icons that play the vibrations associated with the application it represents as they are pressed. Similarly, the icons located at the bottom section provide the sound information about the associated application when they are pressed. When two icons are pressed (one from the top section and the other from the bottom section), the icon associated with the application is shown at the top section, and the name of the application is played as a voice message as well as whether the pairing is correct. If the pairing is correct, the haptic icon at the top section will remain visible, and the icon located at the bottom section will be hidden. On the other hand, if the pairing is wrong, the haptic icon will be replaced by a cross icon, and the icon located at the bottom section will be shown again. As in the reinforcement learning stage, different feedback sounds are played (beeps) depending on the zone of the screen that is pressed (top or bottom zone) (Fig 7).

The HAPP application has two modes of operation that enable an evaluation of the capacity of the subjects to memorize the association between the haptic icons and the applications as a function of the learning process (with and without a reinforcement learning stage).

After the test was completed, all the subjects were required to fill out a usability form composed of six questions. Three of the questions were an adaptation of the System Usability Scale (SUS) and Computer System Usability Questionnaire (CSUQ) [43] usability forms. The other three questions were focused on learning-related aspects: perception, differentiation and recognition [40]. A Likert scale from 1 to 7 (from totally disagree to totally agree) was employed for answering the questions on the evaluation form. Table 1 shows the evaluation questions.

**Table 1:** Usability form.

Question number	Question
1	Vibrations were clearly perceived.
2	Vibrations were distinguishable.
3	It was possible to recognize the meaning of each haptic icon.
4	I would like to assign vibrations to identify application alerts on my own mobile phone.
5	I will need more practise to remember all the vibration patterns.
6	The HAPP application is easy to use.

## Results

In this section, we present the main results of the experiment focusing on the statistically significant results (p < 0.05).

### Test time

The mean time taken to complete the test stage was 4:14 minutes (with a minimum time of 1:48 and a maximum time of 10:43). The analysis of variance (ANOVA) results confirmed the differences in execution time between the VI and non-VI subjects (F(1,42) = 8.01, p<0.01). These differences are due to the time needed to explore the screen to locate the icons to press.

### Recognition rate

**Fig 8:**
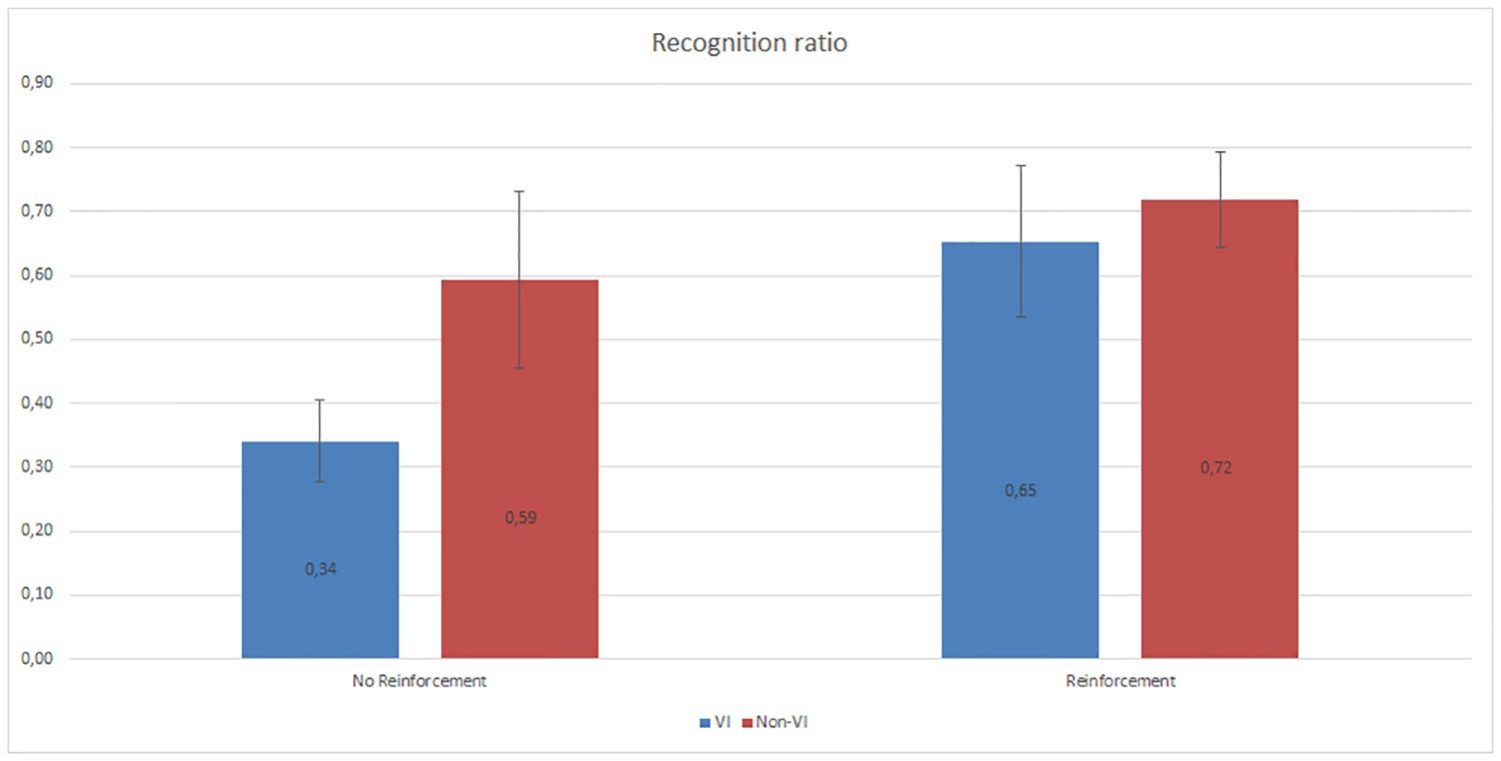
Global haptic icon recognition ratio. For all the subjects, this figure the recognition ratio as a function of the use or not of the reinforcement stage and differentiating VI and non-VI subjects.

The two-way ANOVA study of the percentage of recognized haptic icons during the test stage showed significant differences between subjects with different visual capacities (F(1,42) = 13.33, p<0.05) and learning processes (F(1,42) = 25.00, p<0,001). As shown in Fig 8, the recognition rate improves when the reinforcement stage is applied, especially for the VI subjects. The non-VI subjects present a better recognition rate when the reinforcement stage is not applied compared to the VI subjects, although this difference practically disappears when the reinforcement learning is used (F(1,21) = 1.23, p>0.05).

**Fig 9:**
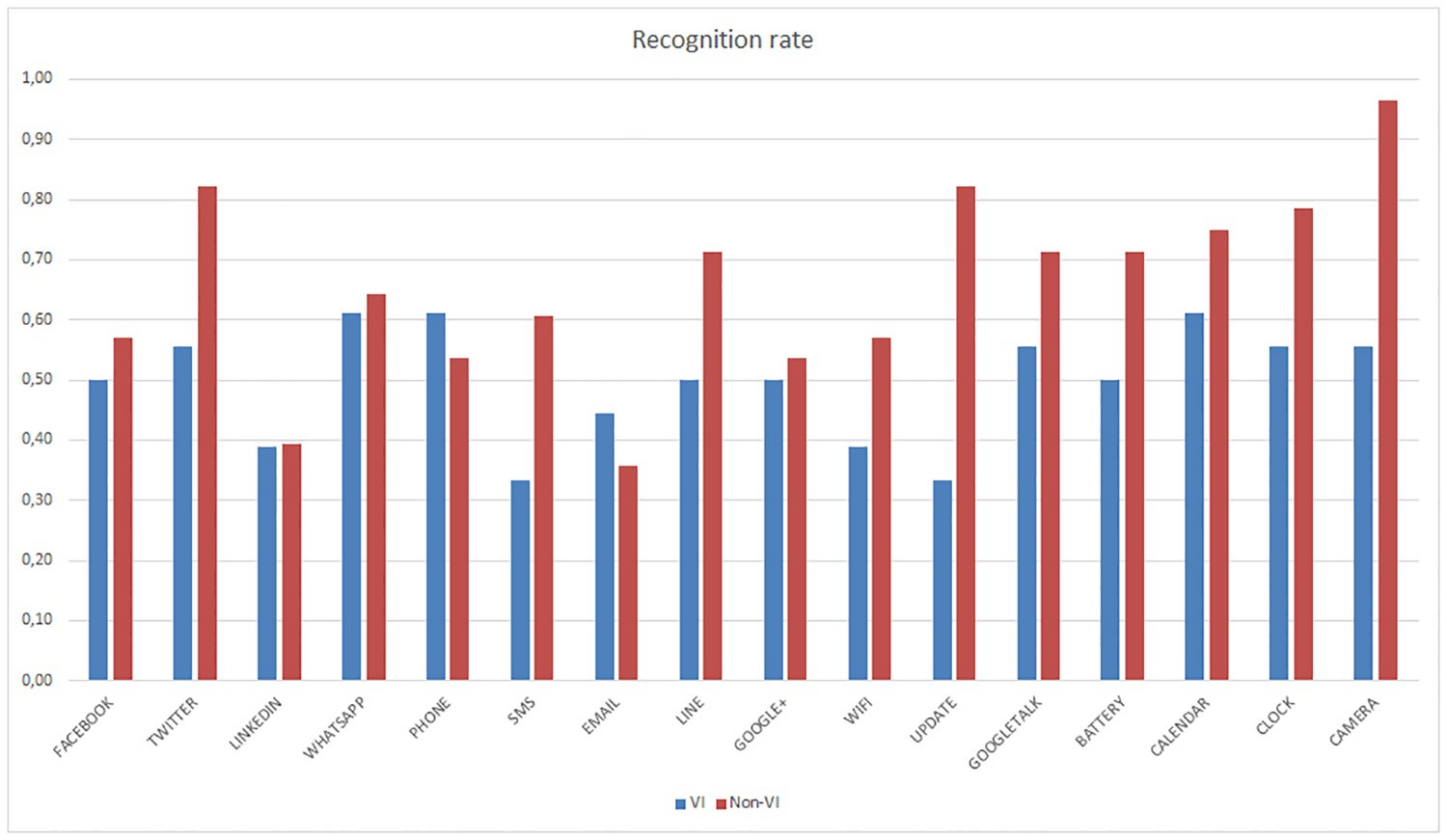
Per haptic icon recognition rate. VI vs Non-VI users for all the population.

**Fig 10:**
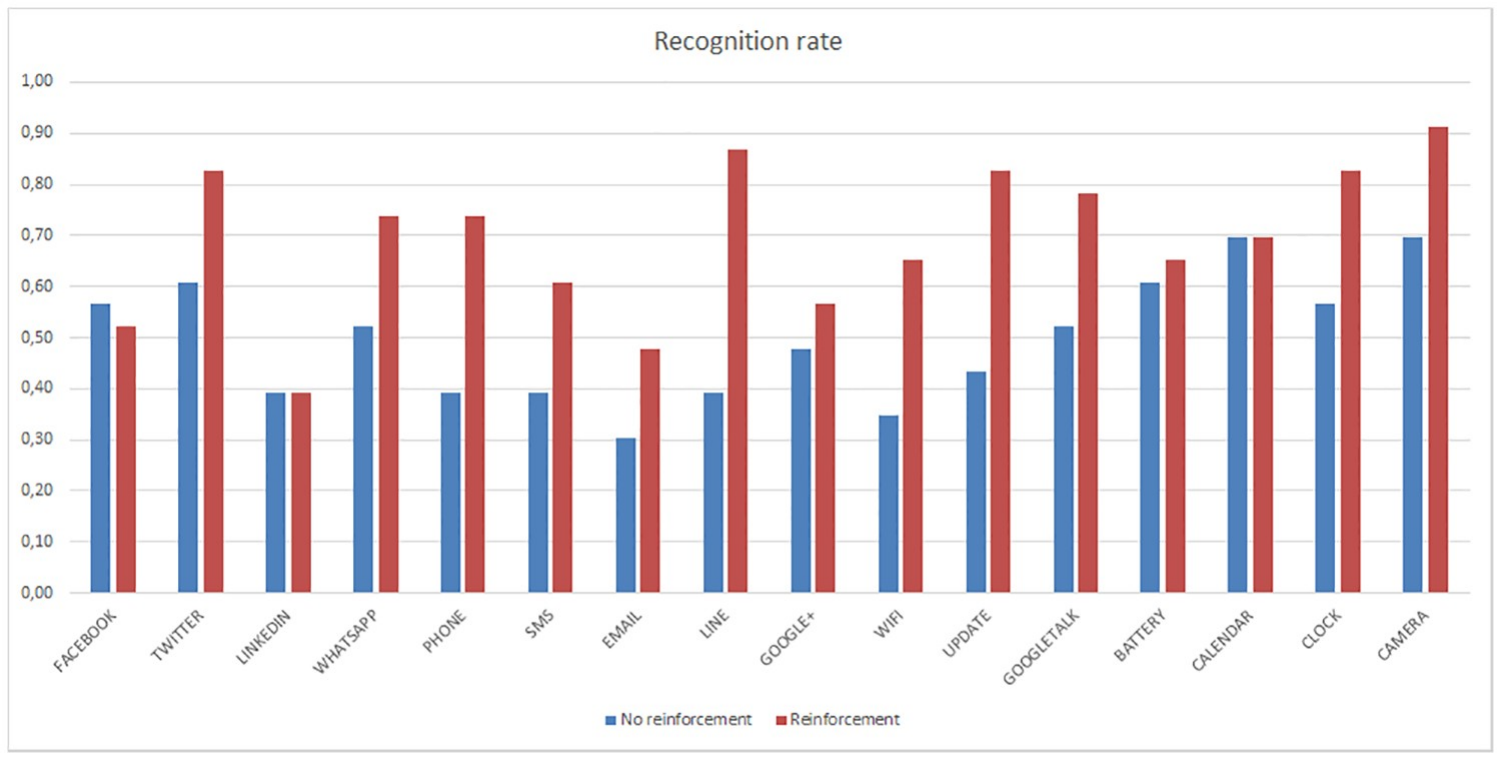
Per haptic icon recognition rate. Reinforcement vs No reinforcement for all the population.

There are significant differences between the recognition rate of the Camera and LinkedIn haptic icons (t_45_ = 4.29, p<0.001) as well as between the recognition rate of the Twitter and Email haptic icons (t_45_ = 3.31, p<0.001). Fig 9 represents the recognition rate of VI and Non-VI subjects without taking into consideration if they used reinforcement or not. Meanwhile, Fig 10 depicts the recognition rate as a function of the reinforcement without taking into consideration if users were VI or not.

Although the obtained similarity map showed that the Twitter, Camera and Calendar haptic icons could be difficult to distinguish, the results show very different behaviour. These three haptic icons obtained recognition rates close to or greater than 0,8, and were the most recognizable icons in some cases, especially when the reinforcement learning is applied. Consequently, the supposition made by [41] that training users could avoid the misrecognition of sforzandos in haptic icons is confirmed.

**Fig 11:**
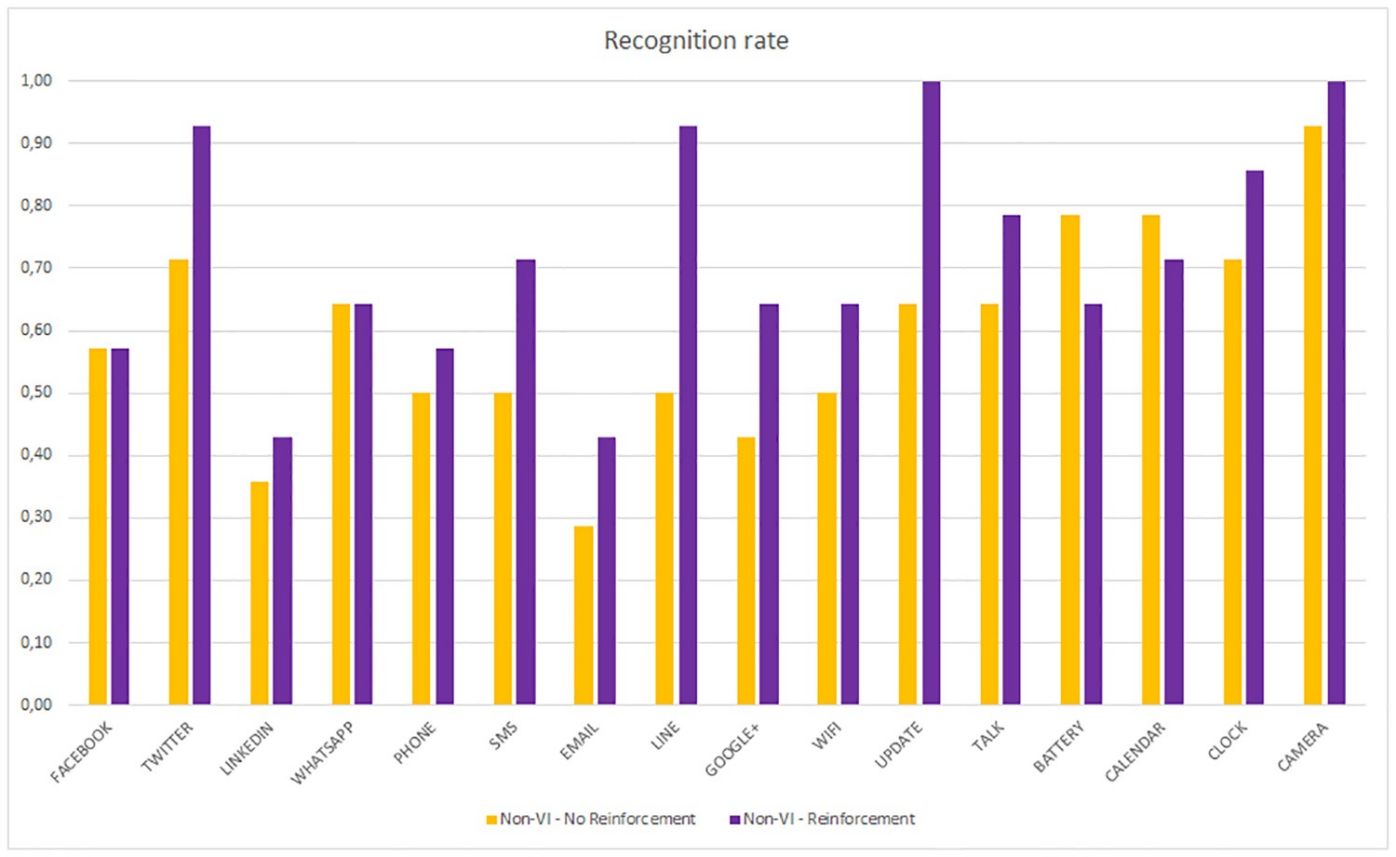
Per haptic icon recognition rate. Non-VI people with and without reinforcement.

Fig 11 shows the recognition rate for each application for the non-VI subjects and compares the results with and without reinforcement learning. Most of the haptic icons have improved recognition rates with reinforcement learning that is maintained, except for the Facebook and WhatsApp applications.

**Fig 12:**
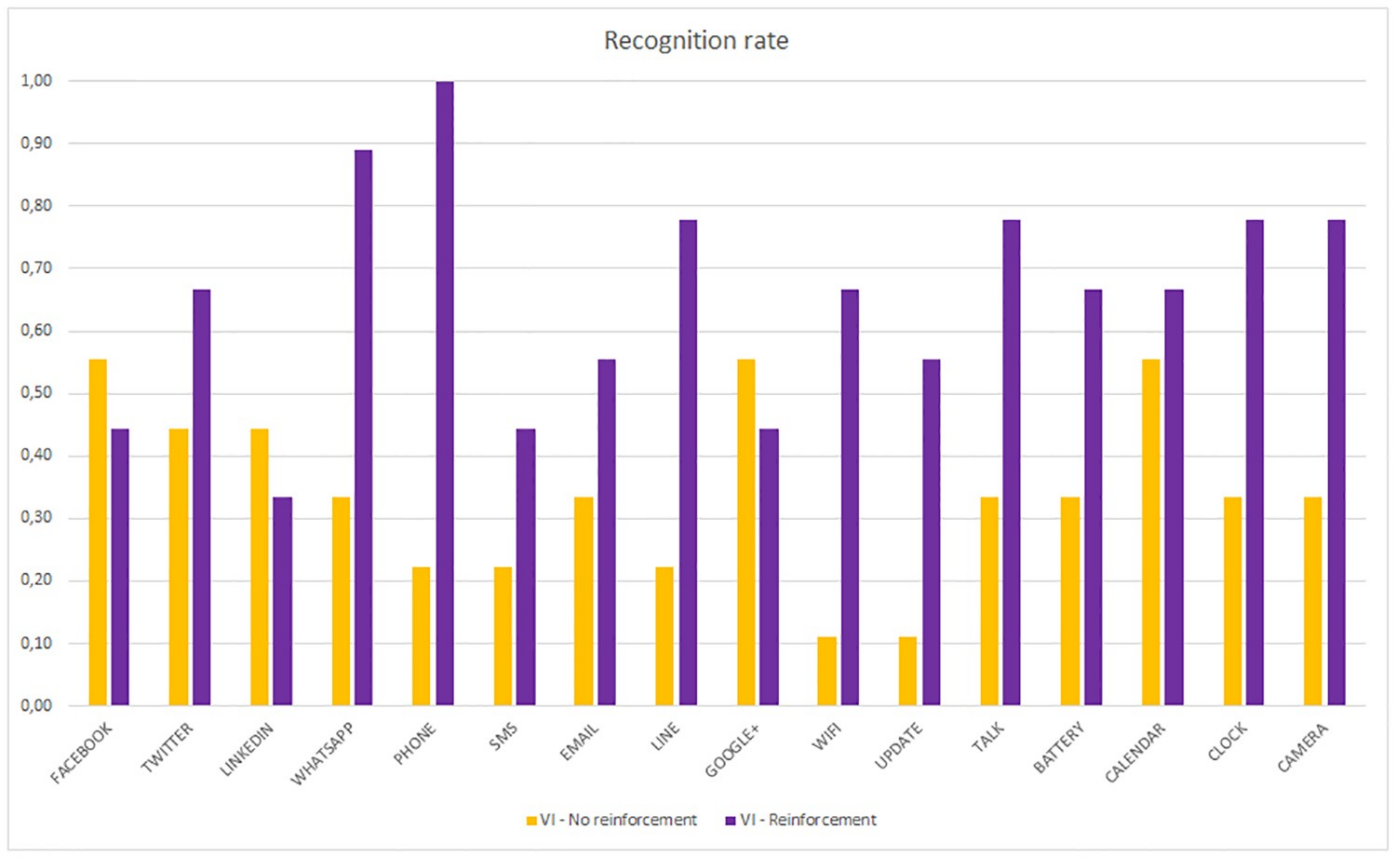
Per haptic icon recognition rate. VI people with and without reinforcement.

Fig 12 represents the recognition rate for each application for the VI subjects with and without reinforcement learning. The improvement obtained by the VI subjects when using the reinforcement stage is very high for almost every application except for Facebook and LinkedIn where the recognition rate decreases.

**Fig 13:**
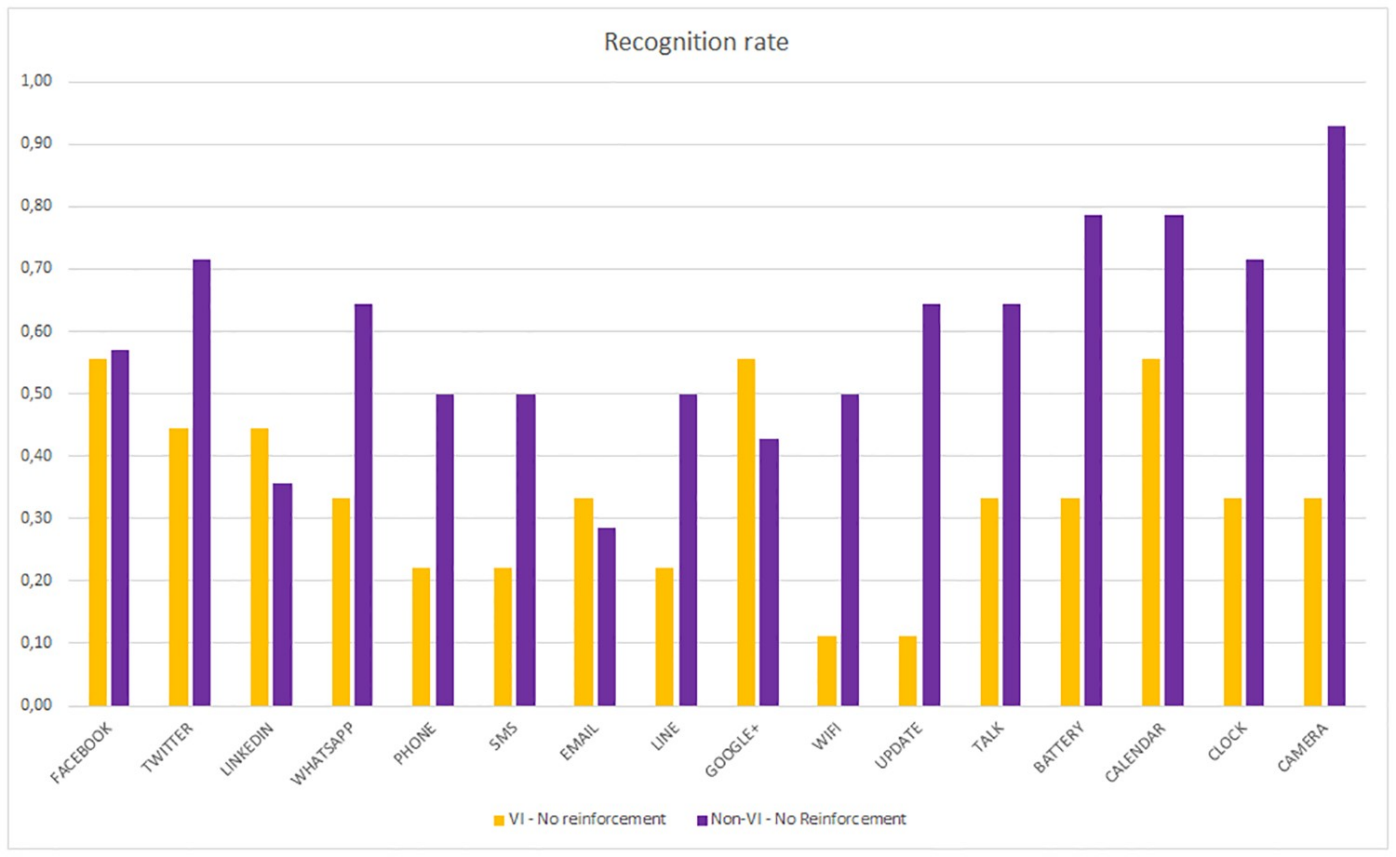
Per haptic icon recognition rate. VI and Non-VI people without reinforcement.

If we compare the recognition rate of the VI and non-VI subjects when no reinforcement is applied, Fig 13 is obtained.

**Fig 14:**
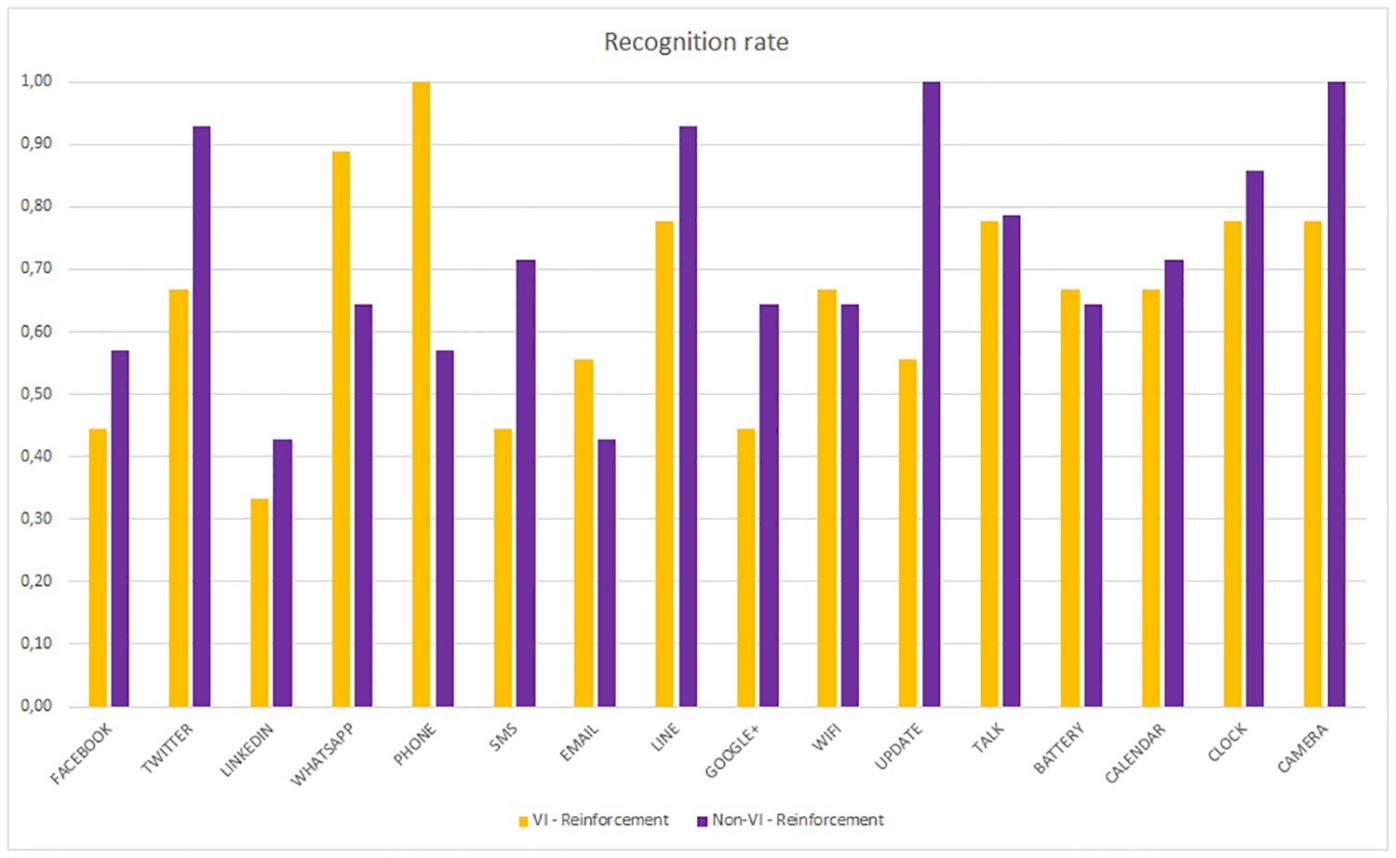
Per haptic icon recognition rate. VI and Non-VI people with reinforcement.

The non-VI subjects obtain a better recognition rate than VI for all applications except for LinkedIn. In some cases, such as Camera, WiFi or Update, the difference is very high. However, once the reinforcement learning is applied, this difference is lower or sometimes the VI subjects obtain a better recognition rate, as shown in Fig 14. The VI subjects who did not use the reinforcement stage obtained the lowest recognition rates (between 11% and 56%). In contrast, the non-VI subjects who did use the reinforcement stage obtained the highest rates (between 40% and 100%).

Both the VI and non-VI subjects improve their recognition rates. Even the applications, such as Twitter, Phone, Line, Update or Camera, obtain rates close to 1.

### Usability form

We performed non-parametric Mann-Whitney U tests to compare the replies to the usability form in terms of the use or not of the reinforcement learning stage for the VI and non-VI subjects.

Considering the reinforcement learning condition, the VI users stated that they were able to better recognize the meanings of the vibrations when the reinforcement learning stage was used (Mann-Whitney U = 17, p<0.05). In contrast, the non-VI subjects did not notice a difference in terms of the reinforcement learning stage (all the answers have p>0.05). However, there were no clear differences in the application usability between the VI and non-VI groups when they used the reinforcement learning stage (p>0.05). However, the visually impaired subjects better distinguished (Mann-Whitney U = 22, p<0.05) and recognized (Mann-Witney U = 28.5, p>0.05) the haptic icons when using the reinforcement stage, although it did not affect the application usability (Mann-Whitney U = 30, p>0.1). Table 2 shows the usability answers from the HAPP application.

**Table 2:** Median of the HAPP application usability form.

	Without reinforcement		With Reinforcement		
Question	Non-VI (n = 14)	VI (n = 9)	Non-VI (n = 14)	VI (n = 9)	Total (n = 46)
1	7	7	7	7	7
2	6	7	5	7	6
3	4	4	4	6	5
4	6	7	7	7	7
5	7	7	7	6	7
6	7	7	7	7	7

## Discussion

In this study, we performed a group of experiments on the learning of a set of haptic icons to identify the alerts for a set of smartphone applications. The results show that learning the set of icons can be performed in less than ten minutes, although this time changes according to the visual capacity of the participant. In the next sections, we discuss the obtained results.

### Design of the haptic icons

We designed sixteen haptic icons and performed a study to evaluate their distinguishability using a multidimensional scaling study. The obtained results showed that they could be easily recognized because the two-dimensional representation of the distances between them was high enough. However, the differentiation test performed by the subjects showed different results, where pairs of applications, such as Twitter and Camera, were difficult to distinguish. This behaviour could be caused by the absence of a learning process, as the test was performed by the subjects who had not previously used the HAPP application. In fact, the recognition rates of those haptic icons (Twitter and Camera) were greater than 0.7 and, in some cases, close to 1.

### Effect on the learning process

Our hypothesis was that a reinforcement learning stage could improve the recognition rate as well as the subjective perception and distinction of the haptic icons. According to the obtained results, we can affirm that the use of a reinforcement stage is positive for the learning process. This claim is derived from the improvement in the recognition rate as well as the answers of the usability form.

### Effect of the subjects’ visual condition

First, it should be noted that the VI and non-VI subjects scored the application usability as high whether or not it was used with the reinforcement learning stage. However, the inclusion of the reinforcement learning stage greatly improved the recognition rate of the blind subjects, making their results as good as the results obtained by the non-VI subjects. Moreover, the inclusion of the reinforcement learning stage also improved the subjective perception of the VI subjects on some particular aspects of application usability. This conclusion is derived from the answers given in the usability form, such as the recognition of the vibration meaning and the distinctiveness of the haptic icons.

There is an interesting effect in this test considering the best and worst recognized icons. The subjects were asked why they had recognized some icons very well but others very badly. Many of them answered that they could easily recognize the icons associated with applications that they used frequently, nevertheless, for the applications that they rarely use or are unknown to them, it was very difficult to identify the associated haptic icon.

## Conclusions

In this paper, haptic icons were employed to identify the alerts generated by applications on a smartphone. Users with a visual impairment greatly improved their recognition rate and their subjective perception of haptic icon recognition when a reinforcement learning stage was added to the learning process.

All the subjects that performed the tests agreed that it was useful to associate different vibrations with the application alerts on the smartphone, although some of the subjects stated that they would only associate the vibrations with those applications that they used frequently. This suggestion is very interesting as it indicates that the utility of the haptic icons could be better if they were applied only to the most commonly used applications or if the user was given a choice to establish a relationship between a haptic icon and an alert.

It should be noted that the subjects did not need much time to learn the associations between the alerts and haptic icons. In addition, the reinforcement learning stage improved the recognition rate of all kinds of users, although this improvement was even better for the VI subjects.

As a future work we propose to test the HAPP application using different smartphones models and test if the recognition rate change due to different perceptions of the vibrations as they may change their intensity and accuracy depending on the smartphone model. Another future development would be to allow the subjects to assign the predefined vibration patterns to the applications and measure if the recognition rate improves.

## Supporting information

S1 File Data files for the perceptual map and recognition per user. The data file contains the raw (Excel and SPSS format) and processed (Excel format) data obtained using the HAPP application per user as well as the individual perceptual map and global perceptual map.
(ZIP)
